# A Record of Wonderful Development

**Published:** 1919-12-27

**Authors:** 


### SURGERY OF THE FRONT IN FRANCE.

### A Record of Wonderful Development.

In Sir Anthony Bowlby's sketch of the "Growth of Surgery of the Front in France," which he delivered to the Abernethian Society of St. Bartholomew's Hospital, the first date of importance is October 13, 1914, the opening day of the "First Battle of Ypres." On this day Sir Anthony arrived at G.H.Q., and applied for leave to stay at the "Front"; up to then a consultant was not expected to proceed forward from the base. The second is November 17, the closing day of the same battle, by which date authority had been got for nurses to be attached to C.C.S., and for beds to be a part of their equipment. Thus it had been recognised that operations done in the forward area were of little value unless the patient could be retained at the spot where the operation was performed and there adequately nursed.

### Neuve Chapelle, Festubert, and Loos.

This was the period in which battles having enormous numbers of casualties began to develop. During it the casualty clearing stations won the confidence of the Army, so that the operative surgery of the forward area was in a position to develop. In the first half of the period the time was spent in training young, capable, and energetic surgeons to do the work in these stations. The Battle of Loos is important in surgical history, for in it an "advanced operating centre" was first used.

### The Somme.

Two new important principles arose out of this battle. The first was that, as consulting surgeon, Sir Anthony Bowlby was told that a battle was going to take place instead of his having to assume it from what he saw; the second was that increased staffs were drafted up to the casualty clearing stations before the operations commenced. The confidence which had been gained in 1915 enabled very great improvements to be made, and as a result large numbers of operations were now done in the forward area. By this time there were five armies instead of one, so that Sir Anthony now had the post of "advising consulting-surgeon" at General Headquarters, and another consulting surgeon was attached to each of the five armies.

### 1917.

This was a year of big offensive operations, entailing enormous casualties—Vimy, Arras, Messines, Passchendaele, Cambrai—and yet so great had been the improvement in the organisation of operative surgery that a far larger proportion of them than in any previous year had been thoroughly treated at the Front. But the year had seen another great step. Up to this stage Sir Anthony's address deals only with operative surgery, and only with work at casualty clearing stations. Now these, although they may come under uncomfortable attention from long-range guns and aeroplane raids, were originally lines of communication units, and remained, we believe, as Army or Corps units until the end. The winter of 1916-1917 was spent in teaching *personnel*, as far forwards as the Divisional units, improved methods of splinting, of resuscitation, and other forms of first aid which had been learnt during the preceding two years. These principles have been put into use during the great fights of the last years.

### March 21 to 28.

Sir Anthony contradicts certain rumours that have arisen with regard to the last great German offensive. He states that not a single patient onc in a C.C.S. was captured, also that the persistet rumour which has spread all over the world that certain C.C.S. was captured at that time with all the staff and nurses is untrue. Every C.C.S. in the Third and Fifth Army area had to retire, but the work which had been done by C.C.S. was taken on by stationary hospitals. Sir Anthony admits, however, that a C.C.S. in the Rheims area was captured in the offensive of May 27. This was serving certain weak British Divisions in this area, and was taken when the line of the Aisne was lost by the Allies at this time.

### The Turn of the Tide.

In the last period of the war Sir Anthony gives rather an epitome of the fighting than any account of the surgery. It appears that the organisation for operative surgery had been so far developed for stationary warfare that further improvements were matters of detail rather than of principle. When the advance began Sir Anthony admits that it was most difficult to attain to the same high surgical ideal; the C.C.S. had to be moved forward, with the result that patients had to be sent off without operation or too soon after operation. Transport began to run short; Sir Anthony says "the C.C.S. must have far more transport allotted to them," but it is just for transport that everyone else is screaming under the same circumstances. We must remember that in France true mobility was never regained before the Armistice. It is of the work in Macedonia, in Palestine, in Mesopotamia, and in German East Africa that we hope the consulting surgeons to these Forces will tell in the way Sir Anthony Bowlby has done of the work in France.

